# Properties of Cavities in Biological Structures—A Survey of the Protein Data Bank

**DOI:** 10.3389/fmolb.2020.591381

**Published:** 2020-11-06

**Authors:** Mateusz Chwastyk, Ewa A. Panek, Jan Malinowski, Mariusz Jaskólski, Marek Cieplak

**Affiliations:** ^1^Institute of Physics, Polish Academy of Sciences, Warsaw, Poland; ^2^Department of Biometry, Warsaw University of Life Sciences, Warsaw, Poland; ^3^Faculty of Physics, University of Warsaw, Warsaw, Poland; ^4^Center for Biocrystallographic Research, Institute of Bioorganic Chemistry, Polish Academy of Sciences, Poznań, Poland; ^5^Department of Crystallography, Faculty of Chemistry, Adam Mickiewicz University, Poznań, Poland

**Keywords:** proteins, cavity volume calculation, plant pathogenesis-related proteins, hydrophobicity, signaling proteins, transport proteins

## Abstract

We performed a PDB-wide survey of proteins to assess their cavity content, using the SPACEBALL algorithm to calculate the cavity volumes. In addition, we determined the hydropathy character of the cavities. We demonstrate that the cavities of most proteins are hydrophilic, but smaller proteins tend to have cavities with hydrophobic walls. We propose criteria for distinguishing between cavities and pockets, and single out proteins with the largest cavities.

## 1. Introduction

Cavities appear in many biological structures (Andrews and Tata, 1971; Martin et al., 1991; Jin and Brennan, 2002; Hartl et al., 2011). Cavities are observed in single-domain proteins (Marion et al., 2007), in multimeric protein aggregates, in virus capsids, (Zandi et al., 2004; Zlotnick, 2005; Michel et al., 2006; Cieplak and Robbins, 2010, 2013; Roos et al., 2010), and in still larger complexes, such as the ribosomes. Biological cavities may enclose space completely, as in the majority of icosahedral virus capsids. Usually, however, the closure is not complete since there are openings or connections to the outside solvent. This situation is encountered, e.g., in the Pathogenesis-Related class 10 proteins (PR-10) (Fernandes et al., 2013). In the ribosome, the opening is a part of the peptide exit channel.

It has been estimated that about 1% of structured proteins are endowed with cavities (Williams et al., 1994). The cavities may or may not be occupied by solvent molecules (Hubbard et al., 1994; Williams et al., 1994), and it is not clear what factors are responsible for that. It is known that in the case of the PR-10 proteins, the cavities serve as reservoirs for small-molecule ligands, but in general, the cavities may play many different roles. For instance, the ribosomal exit channel supports the formation of secondary structures in the nascent proteins, while viral cavities encapsulate, and may help to pack the genomic nucleic acid. The presence of a cavity in thermophilic proteins influences their stability. The stability is also affected by the character of the hydropathy of the internal cavity walls. Hydropathically neutral cavities are expected to prevent reversible protein unfolding, whereas hydrophobically lined cavities destabilize folded structures (Xue et al., 2019).

To gain insights into the properties and roles of protein cavities, we conducted two surveys: (1) of 24,280 single-chain protein structures from the CATH database (Dawson et al., 2017; Lewis et al., 2018) and (2) of all 160,233 structures released by the Protein Data Bank (PDB) (Berman et al., 2000) on February 9, 2020, with 148,516 of them corresponding to proteins without any admixture of nucleic acids. In the former case, we calculated the volume of the cavities within each single chain deposited in the CATH database, but if there were several chains of the same protein, we considered only the case with the largest cavity. In the latter case, we considered all kinds of possible cavities: within the component subunits as well as cavities created within the complete oligomeric structure. We discuss the CATH-based survey separately because these structures are of good quality. The CATH proteins constitute a subset of the full PDB set. The results for all PDB proteins can be found at our website: http://www.ifpan.edu.pl/chwastyk/spaceball. The objective of our studies is to gain an overview of the known protein structures from the point of view of their internal cavities.

Our survey is focused on identifying structures with the largest cavities, and on determining the hydropathy levels of the cavities. There are many hydropathy scales available (Palliser and Parry, 2001; Kapcha and Rossky, 2014). We have chosen the scale constructed by Kyte and Doolittle (1982) as it seems to be the most widely used. The specific hydropathy values that we derive for the cavities are expected to depend on the choice of the scale. However, the relations among the calculated values and the resulting trends are expected not to be very sensitive to the choice.

The cavities of the PR-10 proteins (Fernandes et al., 2013) are usually hydrophobic. This means that they can accommodate hydrophobic ligands as they are excluded from the hydrophilic cytosol. It is not clear, however, whether these proteins are typical or unusual in this respect. Here, we show that the PR-10 proteins represent, in fact, a minority as most protein cavities found by us are hydrophilic. The PR-10 proteins have been well-studied before so they may serve as a benchmark in our studies.

## 2. Materials and Methods

There are many programs and algorithms that allow one to detect, define, and calculate the geometrical parameters of cavities. We discuss them in Chwastyk et al. (2014). All of them have to address the problem of how to delineate a cavity from the external environment of the protein. The choice of the method affects the estimate of the volume of the cavity. In addition, one usually has to start with a visual identification of the location of a cavity. Thus, these methods are not fully objective. Several years ago, we proposed a more objective approach to the problem of cavity-volume determination (Chwastyk et al., 2014, 2016) by using an algorithm that we named SPACEBALL. We define the cavity as a region that is surrounded by atoms into which no water molecule can enter when moving along some straight line from the outside. This definition holds for a static structure but is also valid for any conformation that arises through thermal fluctuations. The size of the cavity depends on the conformation. Thus, thermal averages of the cavity volume can be obtained by considering sets of conformations that correspond to a given temperature.

**Figure 1:**
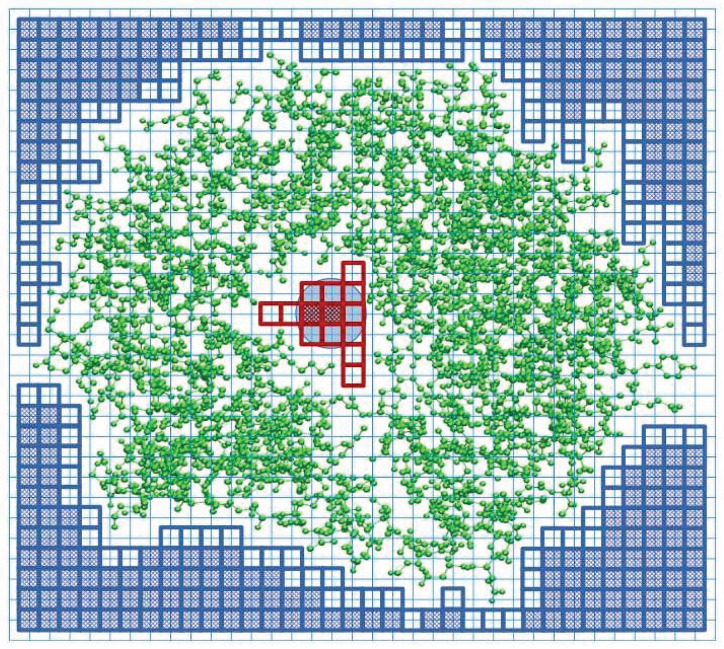
Explanation of the SPACEBALL algorithm used for the determination of the cavity position and volume in a two-dimensional cross-section of a protein with the PDB code 1u8e (green ball-and-stick model). The cavity is part of the white area, covered with the red squares (transparent or shaded). The blue circle represents a water probe. The protein is placed in a cuboid box that is divided into a grid (thin blue lines). The probe is placed at each grid point and we check if there is overlap between the probe and protein atoms. The grid points without any overlap are counted as belonging to the cavity (shaded red squares). We also count the transparent red squares which are encompassed by the probe sitting on the allowed grid points, even if the probe placed on the transparent square would overlap with the protein atoms. The blue squares indicate the lattice points where the probe was placed to define an exterior of the protein. The squares shaded in blue indicate the grid points of the lattice where the probe can be placed without any overlap with the protein atoms. The transparent blue squares indicate the grid points that are encompassed by the probe when it is placed on the allowed blue grid points. The transparent blue squares mark the outer surface of the protein.

In order to detect a cavity in a protein structure, we place the structure in a cuboid box with a regular grid of lattice points, as shown schematically in Figure 1. The default lattice constant is set at *a* = 0.2 Å. Each of the six walls of the box is the source of “rain” of water molecules. The rain is modeled by a network of beads of radius *r*_*w*_ = 1.42 Å corresponding to the water molecules. When a water molecule moves in a given direction it marks the grid points it has visited. It stops when the sphere with the radius *r*_*w*_ overlaps with any of the spheres associated with the atoms of the considered biomolecular structure. The radii of the atomic spheres are taken as the van der Waals radii compiled in the classic book by Pauling (1960). All of the unmarked points define the interior of the structure. In the next step, we put the water-molecule probe on the remaining (not visited) grid points, and check whether the probe does not overlap with the molecular structure. If it does, we count such points as belonging to the structure. The total number of such points, when multiplied by *a*^3^, determines the total volume of the structure, *V*_*T*_. If there is no overlap, such a grid point is counted as belonging to the cavity. The total number of these points multiplied by *a*^3^, determines the total volume of the cavity, *V*_*C*_. If the interior of the structure is divided into separate chambers, then the volume of the largest chamber is taken as representing the cavity volume of the structure.

The accuracy of the results depends on the selection of the lattice constant. Theoretically, the smaller the value of *a*, the more accurate the results but also the lower the efficiency of the calculations. In our previous studies, Chwastyk et al. (2014, 2016) we chose *a* = 0.2 Å. Nevertheless, we found that such a small value of the lattice constant is not optimal in the context of a large-scale survey. Instead, in the present work, we use *a* = 0.6 Å. This value is still smaller than the probe radius *r*_*w*_ = 1.42, so the final result is still correct though somewhat less precise. Previously, we have also showed (Chwastyk et al., 2014, 2016) that to obtain accurate result it is necessary to average the results over a number of rotations of the macromolecule within the box. Instead of the 25 rotations recommended before, we now implement five random rotations for each structure. We found that this approximation is sufficient for the purpose of the present surveys.

Amino acids that are considered as belonging to the cavity shell were selected by calculating the distances between the grid points that define the cavity surface and the surrounding amino acids. The area of the cavity surface was calculated by using the SPACEBALL algorithm but this time for the pseudo-structure created by water molecules placed on the grid points defining the cavity. This allowed us to select the points on the cavity surface. The amino acid in the smallest distance in a given direction was considered as a part of the cavity shell. This procedure used the Python MDAnalysis package (Michaud et al., 2011; Gowers et al., 2016). The grid points without any protein atoms along the line connecting the cavity with the outside of the protein are considered as entrances to the chamber.

All of the results presented in this manuscript were obtained in the CATH-based survey. Only single protein chains were considered. Non-protein parts were removed.

## 3. Results

### 3.1. Geometrical Properties of Cavities

**Figure 2:**
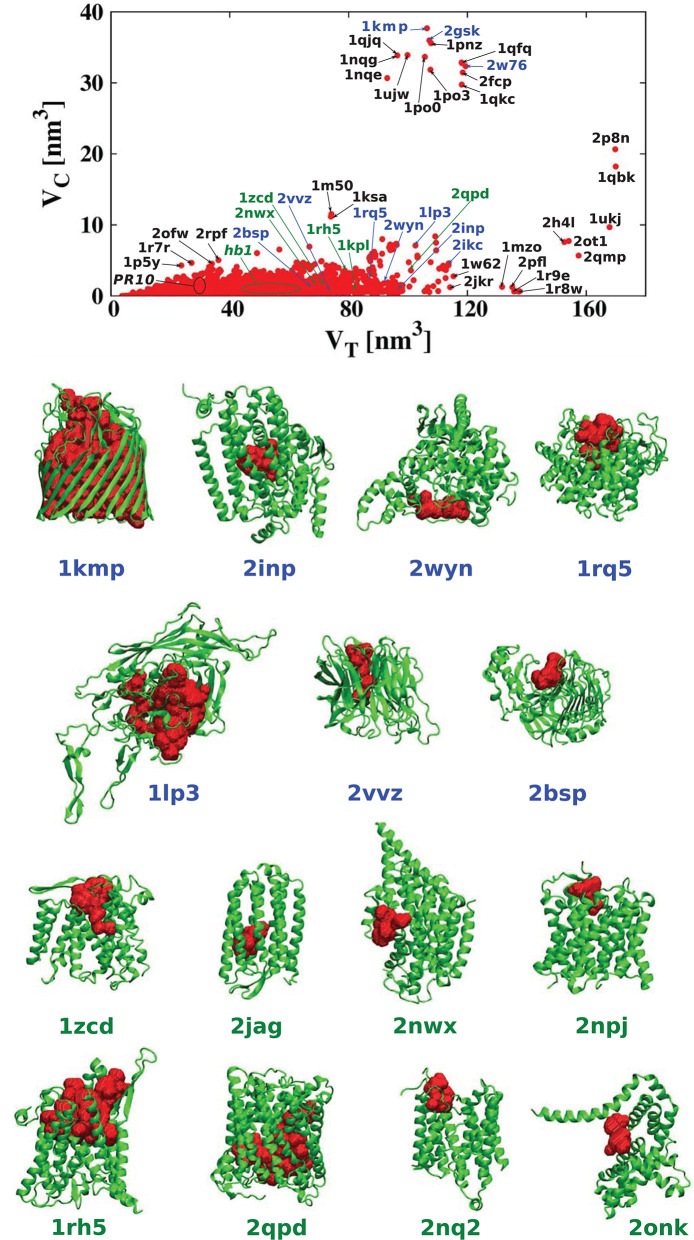
(Top) Volume of the cavities *V*_*C*_ as a function of the total volume *V*_*T*_ of the corresponding proteins. The proteins with the most hydrophobic and the most hydrophilic cavities as well as some outlying structures are marked by their PDB codes. Ten most hydrophobic and ten most hydrophilic structures are marked in green and blue, respectively. Among the most hydrophobic structures there is one group of proteins at similar position in the scatterplot and they have been grouped together and marked by oval (hb1): 2jag (29.76 kDa), 2onk (303.31 kDa), 2nq2 (130.20 kDa), 2npj (44.66 kDa), 2b2h (42.09 kDa). Seven of the most hydrophilic structures and eight of the most hydrophobic structures with different folds are shown in the panels below. The protein structures are in green and the cavities are in red. Nine PR-10 proteins considered separately in this survey are also grouped together in one oval: 2bk0 (32.67 kDa), 2wql (66.84 kDa), 2flh (72.47 kDa), 1txc (34.29 kDa), 1tw0 (32.79 kDa), 1vjh (27.75 kDa), 1qmr (17.33 kDa), 1llt (17.39 kDa), 1xdf (33.85 kDa).

For each of the analyzed proteins, we determined the position of the largest cavity, its volume *V*_*C*_, and we identified the residues that form the cavity shell. Moreover, we calculated the total volume *V*_*T*_ of the whole protein. Figure 2 shows a cross plot of *V*_*C*_ vs. *V*_*T*_. It is seen that large proteins may have both small [1mzo (170.98 kDa), 2pfl (170.77 kDa), 1r9e (176.46 kDa), and 1r8w (176.31 kDa)] and large cavities [for instance, 2p8n (30.49 kDa), 1qbk (127.67 kDa), 2w76 (176.03 kDa), 1qkc (85.15 kDa), 1qjq (84.92 kDa)]. Small proteins, like 1r7r (91.06 kDa) or 1p5y (61.56 kDa), can still have substantial cavities. The panels below the plot, show fifteen most representative structures of the proteins identified in the *V*_*C*_ − *V*_*T*_ plot by their PDB code. The largest chambers of the cavities are marked by red color. They are divided into two groups. The two top lines represent proteins with the most hydrophilic cavities (blue codes), and the bottom lines show structures of the proteins with the most hydrophobic cavities (green codes).

**Figure 3:**
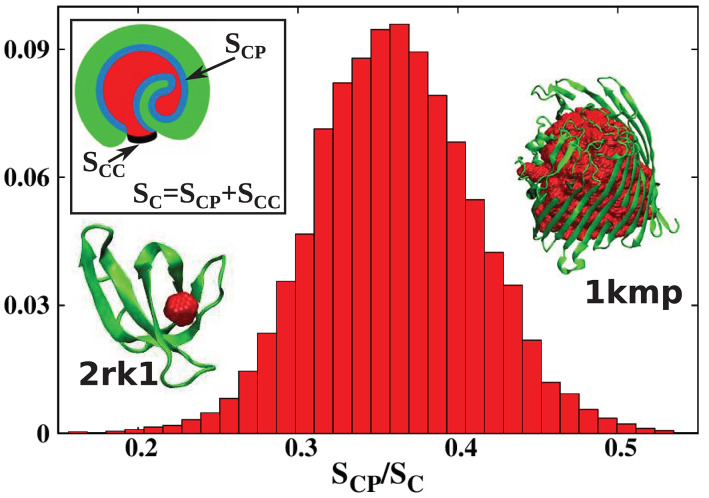
Histogram of the values of the parameter *s* = *S*_*CP*_/*S*_*C*_ obtained in this survey. The *s* parameter defines the degree of cavity closure. The mean(standard deviation) of this parameter is $\bar{s}\left(\sigma \right)=0.36\left(0.05\right)$. Pockets correspond to $s<\bar{s}-3\sigma$ which means *s* < 0.21. The inset shows a schematic representation of *S*_*CP*_ (blue) and *S*_*C*_ (blue combined with black). The protein with the most buried cavity is chain A of the iron(III) dicitrate transport protein Feca [PDB: 1kmp (90.28 kDa)]. The protein with the most open cavity considered in this work is chain A of DHFR R67 Complexed with NADP and dihydrofolate [PDB: 2rk1 (8.27 kDa)].

Usually, the boundary between a cavity and the external environment is not marked by protein atoms, but it is defined by the protein shape. This means that the cavity is open to at least some extent. To distinguish such cases from proper, fully enclosed cavities, we will refer to such formations as pockets. There is no rigorous definition of an internal molecular pocket in the literature. Based on our experience, we propose the following distinction between a pocket and a true cavity. We calculate the fraction *s* = *S*_*CP*_/*S*_*C*_ where *S*_*C*_ is the total surface of the cavity, and *S*_*CP*_ is the surface of sites that are in the immediate contact with the protein. This is illustrated in the inset of Figure 3: *S*_*CP*_ is indicated in blue, and *S*_*C*_ as a combination of blue and black. The black line corresponds to the closing cup of the cavity. The protein is shown in green and the cavity in red. For a Gaussian approximation of the distribution of the *s* values calculated for all structures considered in our survey and presented in Figure 3, we obtained the mean value of $\bar{s}=0.36$ and standard deviation of σ = 0.05. We define pocket as corresponding to the situation where $s<\bar{s}-3\sigma$, i.e., when *s* < 0.21. This criterion means that most of the cavity is exposed to the solvent. The results presented in Figure 3 change our view of cavities in real proteins.

In the literature (Benkaidali et al., 2013), cavities in proteins are defined as a space buried inside the protein, and connected to the outside environment by channels. Some cavities, however, arise very close to the outside protein surface, and are very well-connected with the outside environment. This can be captured by introducing the parameter *s*, described above that is equal to 1 for a closed sphere, and is much smaller for fairly open cavities. We find that there are only thirteen proteins with cavities with *s* > 0.85 and similar folds. They correspond to the points shown at the top of Figure 2. The largest of them had *s* = 0.94, and corresponded to chain A of the iron(III) dicitrate transport protein Feca (PDB: 1kmp). Its structure corresponds to the top leftmost panel of the structures shown in the figure.

To describe the shape of a cavity, we introduce two parameters: *R*_*g*_ and *w* (Cieplak et al., 2014). Here,

(1) $$\begin{array}{r} R_{g}=\frac{1}{N_{C}}\left(\overset{N_{C}}{\underset{k=1}{\sum }}{\vec{r}}_{k}^{2}\right) \end{array}$$

where *N*_*C*_ is the number of cavity-surface residues, and ${\vec{r}}_{k}$ is their position vector with respect to the center of mass of these residues, i.e., protein's amino acids which are in contact with the cavity.

**Figure 4:**
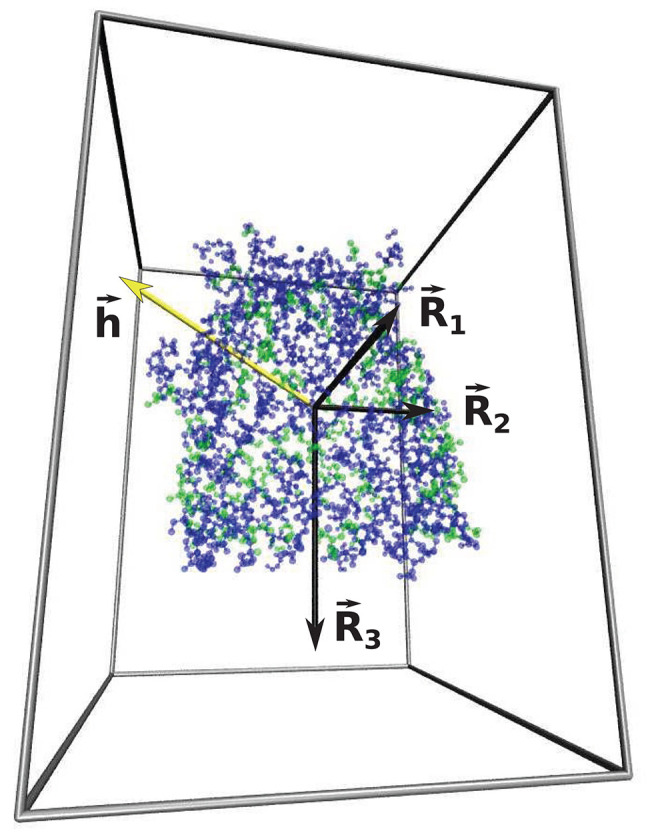
The representation of three main radii ${\vec{R}}_{1}$, ${\vec{R}}_{2}$, ${\vec{R}}_{3}$ (marked by the black arrows) associated with the eigenvalues of the tensor of inertia of 1kmp (90.28 kDa) protein cavity shell. The blue and green dots represent the hydrophilic and hydrophobic amino acids, respectively. The yellow arrow represents the hydropathy vector, $\vec{h}$. The gray lines indicate the calculation box and offers a perspective.

The parameter *w* that characterizes the nature of the shape of the cavity depends on all three main radii, *R*_α_, associated with the eigenvalues of the tensor of inertia (Foote and Raman, 2000) *D*_α_ characterizing the cavity wall: $R_{\alpha }=\sqrt{D_{\alpha }/N_{C}}$, as represented in Figure 4. *R*_1_ is the smallest radius and *R*_3_—the largest. The parameter *w* is defined as

(2) $$\begin{array}{r} w=\frac{\Delta R}{\bar{R}} \end{array}$$

where $\bar{R}=\frac{1}{2}\left(R_{1}+R_{3}\right)$ and $\Delta R=R_{2}-\bar{R}$. Spherical shapes correspond to *w* being close to 0. The tensor of inertia is calculated using all atomic masses of residues belonging to the surface of the cavity. Elongated cigar-like shapes yield substantial positive values of *w* because then *R*_2_ is close to *R*_3_ and $w\sim \frac{1}{2}\left(R_{2}-R_{1}\right)$. Substantial negative values of *w* indicate planar shapes as then *R*_2_ ~ *R*_1_ and $w\sim \frac{1}{2}\left(R_{1}-R_{3}\right)$. The values of the geometrical parameters calculated for 50 structures with neutral, and the most hydrophobic or hydrophilic cavities are presented in Tables 1, 2. The results for all of the calculated structures can be found on our website at http://info.ifpan.edu.pl/chwastyk/spaceball.

**Table 1:** PDB ID, cavity volume *V*_*C*_, surface of sites that are in the immediate contact with the protein *S*_*CP*_, *s* = *S*_*CP*_/*S*_*C*_, where *S*_*C*_ is the total surface of the cavity, parameter *w*, radius of gyration *R*_*g*_ and hydrophobicity *H* for 50 structures with the largest hydrophilic and hydrophobic cavities.

**Hydrophilic**							**Hydrophobic**						
**PDB**	***V*_*C*_ (nm^**3**^)**	***S*_*CP*_ (nm^**2**^)**	***S*_*CP*_/*S*_*C*_**	***R*_*g*_ (nm)**	**w**	**H**	**PDB**	***V*_*C*_ (nm^**3**^)**	***S*_*CP*_ (nm^**2**^)**	***S*_*CP*_/*S*_*C*_**	***R*_*g*_ (nm)**	**w**	**H**
1kmp	37.68	11.23	0.94	26.27	0.27	−381.3	2npj	0.40	2.38	0.35	19.42	0.49	378.0
2w76	32.35	9.74	0.93	27.29	0.50	−378.1	2npc	0.50	3.95	0.40	19.45	0.49	370.4
2inp	0.55	3.77	0.36	23.49	0.71	−373.7	2now	0.71	6.06	0.39	19.52	0.49	368.1
1po0	33.66	9.74	0.89	26.09	0.27	−369.0	1xqf	0.48	4.37	0.42	19.77	0.49	366.8
1kmo	18.98	11.14	0.87	26.30	0.27	−368.3	1xqe	0.38	4.00	0.45	19.34	0.51	363.5
1po3	19.53	9.53	0.92	26.14	0.63	−366.7	2nwx	0.59	3.94	0.34	21.69	0.24	356.5
1lp3	7.11	26.88	0.26	28.50	0.59	−365.9	1rh5	3.40	15.57	0.30	22.82	0.59	350.4
2ikc	4.32	23.77	0.31	23.96	0.07	−364.5	2nww	1.00	6.09	0.33	21.82	0.22	341.9
1pnz	35.58	10.48	0.90	26.40	0.27	−363.0	1u77	0.73	4.64	0.31	19.60	0.62	341.8
2wyn	1.34	5.63	0.38	22.76	0.59	−359.6	1u7c	0.66	3.95	0.35	19.61	0.62	341.7
2vvz	0.62	3.96	0.35	19.92	0.74	−357.7	1zcd	1.24	7.77	0.34	20.79	0.75	337.8
2qpk	3.65	16.96	0.30	23.83	0.28	−355.4	2qpd	3.61	18.14	0.32	23.06	0.32	336.3
2gsk	19.97	45.46	0.21	25.47	0.33	−354.4	1u7g	0.60	4.30	0.37	19.60	0.62	331.1
1rq5	1.63	8.17	0.31	21.22	0.66	−352.7	2nq2	0.64	4.09	0.38	19.66	0.44	325.2
2ips	4.58	24.86	0.31	23.92	0.28	−352.6	2onk	0.85	3.06	0.30	22.24	0.75	302.4
2bsp	0.82	5.64	0.34	20.88	0.62	−352.1	1kpl	0.93	5.80	0.32	22.87	0.61	294.7
2x2h	1.18	10.34	0.33	22.54	0.59	−351.2	2b2h	0.88	4.83	0.30	19.88	0.12	286.8
2nqx	4.06	16.96	0.31	23.75	0.28	−348.7	2exw	0.94	4.83	0.33	22.18	0.52	269.0
2eha	4.04	17.23	0.31	23.78	0.53	−348.2	2fed	0.94	6.68	0.40	22.16	0.51	264.2
2pum	3.81	19.55	0.33	23.76	0.28	−348.0	1kpk	1.01	4.33	0.27	22.79	0.74	263.7
1oyg	0.60	2.69	0.34	20.80	0.51	−347.7	2jag	0.51	2.30	0.28	18.70	0.48	240.9
1ujw	33.92	10.28	0.89	25.70	0.69	−345.9	1ldf	1.06	6.23	0.36	18.15	0.84	231.0
1tz7	2.91	13.17	0.30	23.11	0.69	−345.4	2ksy	0.63	3.32	0.29	17.87	0.10	230.2
2jjb	0.82	3.42	0.29	22.56	0.72	−344.9	2abm	0.34	2.39	0.42	16.59	0.49	229.8
1nqg	33.87	10.17	0.92	25.48	0.54	−344.0	1jgj	0.83	5.07	0.35	17.45	0.62	228.1
1pt2	0.61	2.58	0.32	20.80	0.51	−343.1	1lda	0.91	4.07	0.32	17.98	0.85	227.4
2vk5	0.76	4.04	0.36	19.44	0.69	−342.8	2f95	0.53	3.57	0.37	17.54	0.71	221.2
2qx2	1.12	9.74	0.41	22.94	0.47	−339.6	1ldi	0.77	2.74	0.33	17.97	0.85	220.8
2jf4	0.83	4.39	0.35	22.57	0.58	−338.4	2vt4	1.74	8.41	0.31	20.07	0.66	220.0
1qlg	0.92	4.48	0.30	18.91	0.20	−337.4	1uaz	0.51	2.32	0.28	18.26	0.78	211.7
2r5l	4.05	17.80	0.30	23.80	0.27	−336.7	1rc2	0.42	2.29	0.39	16.98	0.51	206.3
2bf6	0.74	4.39	0.36	19.46	0.69	−336.5	1orq	1.36	8.37	0.36	24.41	0.83	205.2
2efb	3.99	16.62	0.28	23.80	0.07	−336.1	2o9g	0.44	1.67	0.39	17.09	0.24	204.8
1qkc	29.76	9.10	0.93	27.24	0.67	−334.8	1q5i	0.55	2.97	0.29	18.01	0.64	204.7
1qfg	32.78	10.13	0.93	27.27	0.68	−334.3	2r6g	0.55	3.68	0.35	20.83	0.64	202.6
2eae	0.43	3.67	0.32	22.31	0.52	−333.8	2wjl	0.47	2.78	0.34	17.88	0.43	202.0
1qjq	32.86	10.36	0.93	27.21	0.68	−330.2	1jv6	0.49	2.34	0.29	17.33	0.55	201.4
1k72	0.52	3.37	0.28	20.42	0.08	−328.3	1s52	0.51	2.61	0.28	17.97	0.64	199.7
1poo	0.90	4.28	0.30	18.88	0.20	−327.6	1kgb	0.48	2.69	0.35	17.84	0.43	199.5
1ktw	2.07	11.46	0.26	23.23	0.61	−327.5	1o0a	0.44	2.48	0.32	17.70	0.43	198.8
2fcp	31.46	9.34	0.93	27.27	0.68	−326.6	1m0m	0.56	3.48	0.35	17.84	0.43	198.8
2vk7	0.91	5.71	0.35	19.57	0.18	−321.2	1kg9	0.45	2.21	0.30	17.72	0.43	198.8
1kb0	0.74	2.66	0.28	22.21	0.50	−320.9	1x0i	0.53	2.67	0.32	17.63	0.55	198.4
1kfg	0.41	3.12	0.31	20.41	0.08	−317.4	2o9d	0.43	1.83	0.39	17.11	0.47	197.4
1xvd	0.65	5.00	0.36	23.93	0.78	−313.1	1ucq	0.47	2.20	0.28	17.94	0.53	197.4
1nqe	30.67	8.67	0.90	25.34	0.52	−311.5	1mgy	0.49	2.14	0.28	17.80	0.53	196.3
1v08	0.94	3.53	0.31	21.40	0.58	−310.0	2vpy	0.48	3.58	0.40	19.40	0.87	196.2
2xcy	0.88	5.08	0.34	19.48	0.66	−310.0	1jv7	0.51	3.50	0.38	17.53	0.54	196.2
2bmh	3.73	18.72	0.31	22.51	0.76	−308.9	2i1x	0.46	2.60	0.33	17.82	0.43	195.7
1orw	6.85	21.01	0.22	24.24	0.60	−308.1	1m0l	0.46	2.28	0.31	17.82	0.43	195.0
**PR-10**													
1llt	1.49	8.10	0.34	15.63	0.56	−54.8	2bk0	2.05	9.37	0.31	15.27	0.77	27.9
1qmr	1.33	6.49	0.31	15.59	0.54	−50.4	2wql	1.88	8.98	0.32	15.19	0.73	26.4
1xdf	0.29	2.14	0.37	15.30	0.31	−46.6							
1vjh	0.45	3.73	0.39	14.93	0.23	−34.9							
1tw0	1.73	9.68	0.37	15.28	0.41	−25.7							
1txc	1.69	8.86	0.32	15.44	0.52	−21.9							
2flh	0.81	3.86	0.30	14.76	0.48	−16.1							

*Data for PR-10 proteins are appended at the end of the table*.

**Table 2:** PDB ID, cavity volume *V*_*C*_, surface of sites that are in the immediate contact with the proteins *S*_*CP*_, *s* = *S*_*CP*_/*S*_*C*_, where *S*_*C*_ is the total surface of the cavity, parameter *w*, radius of gyration *R*_*g*_ and hydrophobicity *H* for 50 structures with hydrophaty index of cavities |*H*| ≤ 0.2.

**Neutral**						
**PDB**	***V*_*C*_ (nm^**3**^)**	***S*_*CP*_ (nm^**2**^)**	***S*_*CP*_/*S*_*C*_**	***R*_*g*_ (nm)**	**w**	**H**
2hcr	0.11	0.71	0.39	14.00	0.84	−0.2
2pby	0.08	0.48	0.31	13.90	0.76	−0.2
2vje	0.04	0.37	0.37	11.05	0.69	−0.2
1kuv	1.02	5.69	0.35	15.19	0.38	−0.1
1m0o	0.35	2.50	0.34	19.05	0.64	−0.1
1n9c	0.44	2.88	0.33	10.54	−0.01	−0.1
1ptf	0.04	0.30	0.32	11.71	0.31	−0.1
1qg6	1.37	6.16	0.28	17.81	0.47	−0.1
1v8b	0.15	0.98	0.36	14.70	0.69	−0.1
1yji	0.28	2.38	0.33	12.69	0.01	−0.1
2ab8	0.03	0.36	0.34	10.33	0.84	−0.1
2adf	0.06	0.79	0.37	13.88	0.78	−0.1
2f2e	0.37	2.29	0.42	13.74	0.68	−0.1
2grv	0.58	3.55	0.32	17.30	0.42	−0.1
2ntf	0.11	0.56	0.37	14.07	0.63	−0.1
2nwu	0.09	0.77	0.38	14.84	0.62	−0.1
2ojz	0.07	0.52	0.33	14.03	0.82	−0.1
2ok0	0.10	1.04	0.45	13.69	0.85	−0.1
1jtv	1.73	7.94	0.32	19.70	0.07	0.0
1lk5	0.12	1.10	0.39	12.61	0.81	0.0
1lk7	0.10	1.12	0.40	12.64	0.81	0.0
1nse	0.05	0.52	0.45	12.94	0.73	0.0
1p6l	0.05	0.38	0.33	12.93	0.73	0.0
1p6m	0.05	0.48	0.47	12.94	0.73	0.0
1qzz	0.18	0.81	0.43	15.17	0.57	0.0
1rs8	0.05	0.33	0.31	12.91	0.73	0.0
1u1t	0.10	0.79	0.33	11.38	0.63	0.0
1uj4	0.17	2.66	0.46	13.12	0.45	0.0
1uj6	0.13	2.15	0.38	13.12	0.46	0.0
2gd1	0.10	0.68	0.39	16.31	0.53	0.0
2hqi	0.04	0.39	0.33	11.00	0.29	0.0
2hux	0.21	1.04	0.32	15.15	0.81	0.0
2hx2	0.05	0.44	0.39	12.96	0.73	0.0
1lfl	0.80	4.16	0.32	15.17	0.82	0.1
1lfv	0.79	4.74	0.34	15.33	0.71	0.1
1liw	0.27	1.50	0.30	12.34	0.57	0.1
1nqo	0.11	0.93	0.35	16.26	0.79	0.1
1p9n	0.62	4.06	0.38	14.89	0.78	0.1
1qsh	0.78	4.15	0.33	15.21	0.47	0.1
1sdk	0.77	4.43	0.38	15.14	0.66	0.1
1sdl	0.71	3.52	0.33	15.18	0.66	0.1
1uiw	0.87	4.85	0.35	15.47	0.61	0.1
1v8p	0.14	1.16	0.38	14.94	0.82	0.1
1vwt	0.77	4.65	0.36	15.27	0.46	0.1
1xxt	0.78	4.31	0.33	15.18	0.57	0.1
2jdk	0.03	0.27	0.32	13.80	0.21	0.1
2pcg	0.08	0.54	0.38	13.12	0.83	0.1
2pju	0.06	0.55	0.36	12.19	0.84	0.1
2put	0.21	1.32	0.35	14.30	0.83	0.1
2vgf	0.20	1.37	0.31	12.41	0.56	0.1

### 3.2. Chemical Properties of Cavities

In the first approach we calculated the degree of cavity hydrophobicity, *H*, in analogy of how it is done for the whole proteins (Cieplak et al., 2014) except that now we consider only the residues that are on the surface (forming the wall) of the cavity. Specifically,

(3) $$\begin{array}{r} H=\overset{N_{CP}}{\underset{i=1}{\sum }}q_{i}, \end{array}$$

where *q*_*i*_ is the hydropathy index of residue *i*, and *N*_*CP*_ is the total number of residues that create the shell. We used *q*_*i*_ values as determined by Kyte and Doolittle (1982).

Moreover, we define the hydropathy vector, $\vec{h}$, of a cavity shell similarly to Cieplak et al. (2014) but again by taking only the shell residues into account:

(4) $$\begin{array}{r} \vec{h}=\overset{N}{\underset{i=1}{\sum }}q_{i}{\vec{\delta }}_{i}, \end{array}$$

where ${\vec{\delta }}_{i}$ is a position vector with respect to the center of mass of the cavity shell. The hydropathy vector calculated for 1kmp (90.28 kDa) protein cavity shell is presented by the yellow arrow in Figure 4.

**Figure 5:**
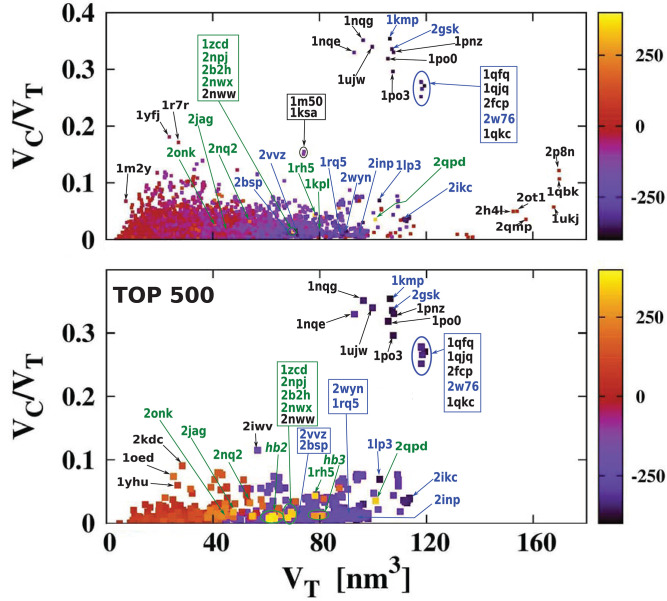
The ratio *V*_*C*_/*V*_*T*_ as a function of *V*_*T*_. It is color-coded to indicate the level of overall hydrophobicity. The bottom plot is similar to the top one, but it shows only the 500 most hydrophobic and 500 most hydrophilic cavities. The H bars on the right define the color code for cavity hydrophobicity. The proteins at similar position in the scatterplot have been grouped together and marked by ovals: hb2: 1u7c (40.47 kDa), 1u77 (40.43 kDa), 1xqe (43.97 kDa), 1xqf (43.87 kDa), 2now (44.79 kDa), 2npc (44.58 kDa), 2npj (44.66 kDa), 2b2h (42.09 kDa); hb3: 2nww (134.64 kDa), 2nwx (135.23 kDa), 1kpk (302.34 kDa), 1kpl (203.18 kDa), 2exw (194.60 kDa), 2fed (193.14 kDa). The most hydrophilic proteins are marked in blue and the most hydrophobic proteins are marked in green.

**Figure 6:**
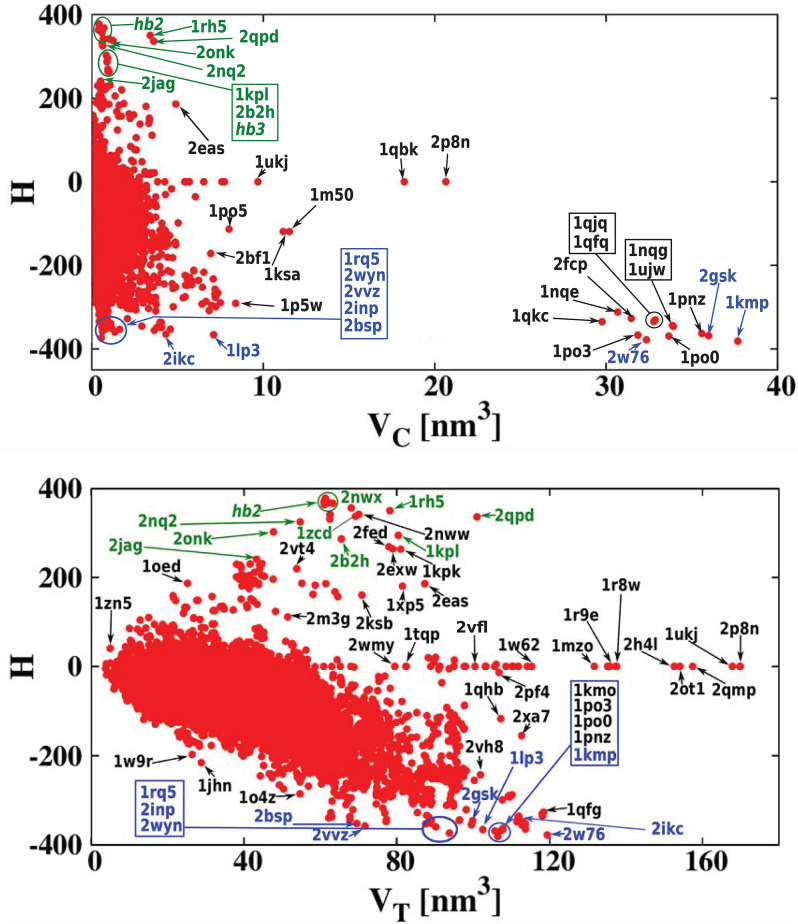
(Top) Cavity hydrophobicity as a function of *V*_*C*_ for all examined proteins. The largest cavities are the most hydrophilic ones. (Bottom) Cavity hydrophobicity as a function of *V*_*T*_ (total volume of protein) for all examined proteins. The most hydrophobic and hydrophilic structures, listed in Tables 3, 4 are marked in green and blue, respectively.

Figure 5 presents the results using a color code for cavity hydrophobicity. The scatterplots present the hydrophobicity of cavities of all structures but considering the thickness of the cavity shell defined as *V*_*C*_/*V*_*T*_. We see that the proteins with the most hydrophilic cavities are those with the biggest cavities which constitute their total interiors. Scatterplots that present explicitly the value of the cavity hydrophobicity in function of the cavity volume or the volume of the whole protein are presented in Figure 6. We see in the scatterplot at the bottom that there are big proteins with neutral cavities [for example: 2p8n (30.49 kDa), 1ukj (172.09 kDa), 2qmp (22.91 kDa), 2ot1 (158.57 kDa), 2h4l (50.75 kDa), 1r9e (176.46 kDa)] or with hydrophilic cavity [for example: 2w76 (176.03 kDa), 1qfg (85.03 kDa), 2qpk (71.76 kDa)] but no big structures with hydrophobic cavities. The scatterplot at the top shows that the largest hydrophilic cavities [for example: 1qfq (8.86 kDa), 1qjq (84.92 kDa), 1qkc (85.15 kDa), 2w76 (176.03 kDa)] are much bigger then the largest hydrophobic and neutral ones [for example: 2p8n (30.49 kDa) or 1qbk (127.67 kDa)]. The detailed results are presented in Tables 1, 2.

**Figure 7:**
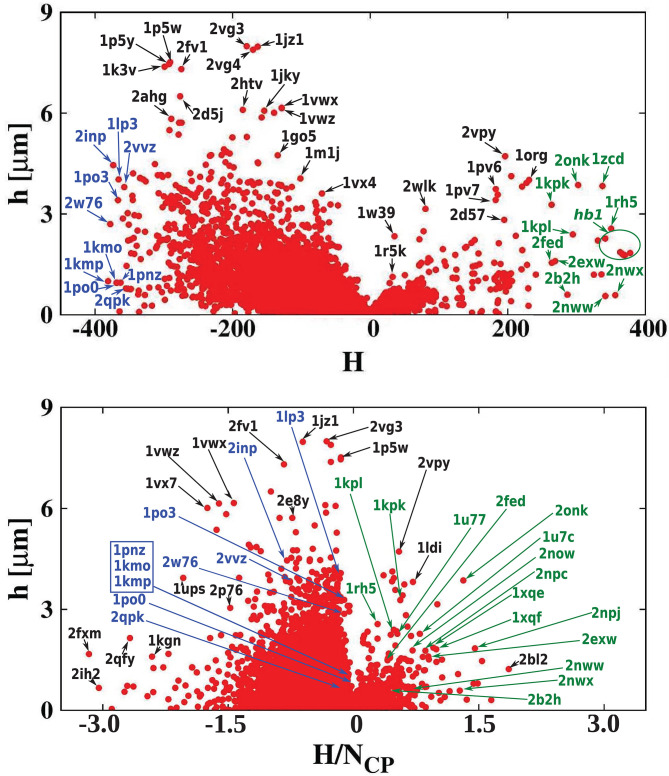
(Top) Absolute value of the hydropathy vector as a function of cavity hydrophobicity for all examined proteins. The most hydrophilic and hydrophobic structures are marked in blue and green, respectively. (Bottom) A similar scatterplot but as a function of cavity hydrophobicity per residue of the cavity surface.

Figure 7 shows the homogeneity of the hydrophobicity of the considered cavities. The scatterplot shows the absolute value of the hydropathy vector $\left|\vec{h}\right|$ as a function of cavity hydrophobicity. As expected, the biggest values of the hydropathy vectors (indicating large hydropathy gradients across the cavity) are found mostly for proteins with hydrophilic cavities. This suggests that the strongly hydrophilic cavities are important for signal transduction (Harley et al., 1998).

## 4. Discussion

We start our analysis of the hydrophobicity of cavities in the examined proteins by considering 10 most hydrophobic and 10 most hydrophilic cavities. The selected proteins are listed in Tables 3, 4, respectively.

**Table 3:** Ten proteins with the most hydrophobic cavities.

**Name**	**PDB**	**H**	**Biochemical function**	**Biological process**	**Cellular component**	**References**
Ammonia channel *Escherichia coli*	2npj (2.00 Å) 44.66 kDa	378.0	Identical protein binding, protein binding, uniporter activity, ammonium transmembrane, transporter activity	Nitrogen utilization carbon dioxide transport ammonium transport ammonium transmembrane transport	membrane plasma	Javelle et al., 2006
Proton glutamate symport protein *Pyrococcus horikoshii*	2nwx (3.29 Å) 135.23 kDa	356.5	Identical protein binding metal ion binding glutamate: sodium symporter activity symporter activity L-aspartate transmembrane transporter activity chloride transmembrane transporter activity amino acid: sodium symporter activity	Protein homotrimerization L-aspartate import across plasma membrane L-aspartate transmembrane transport L-glutamate transmembrane transport aspartate transmembrane transport chloride transmembrane transport amino acid transport	membrane plasma	Boudker et al., 2007
Preprotein translocase secY subunit *Methanocaldococcus jannaschii*	1rh5 (3.20 Å) 61.93 kDa	350.4	Protein transmembrane transporter activity	Protein transport	membrane plasma	Berg et al., 2004
*Na*^+^/ *H*^+^ antiporter protein *Escherichia coli*	1zcd (3.45 Å) 82.78 kDa	337.8	Cardiolipin binding sodium: proton antiporter activity antiporter activity sodium ion transmembrane transporter activity	Regulation of intracellular pH response to cation stress sodium ion export across plasma membrane cellular sodium ion homeostasis sodium ion transport ion transport	Membrane plasma	Hunte et al., 2005
Cytochrome c oxidase polypeptide *Thermus thermophilus*	2qpd (3.25 Å) 87.51 kDa	336.3	Metal ion binding	Oxidation-reduction process	Membrane plasma respirasome	Liu et al., 2007
ABC transporter ATP-binding protein HI1470 *Haemophilus influenzae*	2nq2 (2.40 Å) 130.20 kDa	325.2	Protein binding	Transmembrane transport ion transport	Membrane plasma	Pinkett et al., 2007
ABC transporter ATP-binding protein ModBC-A *Archaeoglobus fulgidus*	2onk (3.10 Å) 303.31 kDa	302.4	Protein binding	Transmembrane transport inorganic anion transport	Membrane plasma	Hollenstein et al., 2007
Chlorine transport protein *Salmonella enterica*	1kpl (3.00 Å) 203.18 kDa	294.7	Chloride ion binding proton antiporter activity antiporter activity chloride transmembrane transporter activity voltage-gated chloride channel activity	Ion transport nitrate transport chloride transport proton transmembrane transport chloride transmembrane transport transmembrane transport	Membrane plasma	Dutzler et al., 2002
Ammonium transporter *Archaeoglobus fulgidus*	2b2h (1.54 Å) 42.09 kDa	286.8	Protein transmembrane transporter activity	Ammonium transport	Membrane	Andrade et al., 2005
Halorhodopsin *Halobacterium salinarumi*	2jag (1.93 Å) 29.76 kDa	240.9	Photoreceptor activity	Response to stimulus ion transmembrane transport protein-chromophore linkage phototransduction ion transport	Membrane plasma	Gmelin et al., 2007

*The first column shows the protein name and its source organism (italicized); the second column lists the PDB codes of particular proteins, the resolution of the structure determination (in parentheses) and the total structure weight; the third column lists the degree of cavity hydrophobicity; the fourth, fifth, and sixth columns report main biochemical function, main biological function and the protein localization in the cell, respectively. The last column lists the references for the presented data*.

**Table 4:** Similar to Table 3 but for 10 most hydrophilic cavities.

**Name**	**PDB**	**H**	**Biochemical function**	**Biological process**	**Cellular component**	**References**
Iron(III) dicitrate transport protein Feca *Escherichia coli*	1kmp (2.50 Å) 90.28 kDa	−381.3	Signaling receptor activity siderophore uptake transmembrane transporter activity	Siderophore transport	Outer membrane	Ferguson et al., 2002
Ferripyoverdine receptor *Pseudomonas aeruginosa*	2w76 (2.80 Å) 176.03 kDa	−378.1	Signaling receptor activity siderophore uptake transmembrane transporter activity	Iron ion homeostasis siderophore transmembrane transport siderophore transport ion transport pyoverdine biosynthetic process	Outer membrane membrane	Greenwald et al., 2009
Phenol hydroxylase component *Pseudomonas stutzeri*	2inp (2.30 Å) 229.82 kDa	−373.7	Oxidoreductase activity	Oxidation–reduction process	Plasma	Sazinsky et al., 2006
Adeno-associated virus (AAV-2) protein *Adeno-associated virus-2*	1lp3 (3.00 Å) 58.77 kDa	−365.9	Structural molecule activity	Permeabilization of host organelle membrane involved in viral entry into host cell viral entry via permeabilization of inner membrane host cell nucleolus clathrin-dependent endocytosis of virus by host cell virion attachment to host cell structural molecule activity	Icosahedral viral capsid	Xie et al., 2002
Sheep lactoperoxidase protein *Ovis aries*	2ikc (3.25 Å) 140.41 kDa	−364.5	Thiocyanate peroxidase activity heme binding metal ion binding	Response to oxidative stress defense response to bacterium	Milk lactoperoxidase (extracellular region)	Sheikh et al., 2006
Protein of periplasmic trehalase *Escherichia coli*	2wyn (2.10 Å) 246.82 kDa	−359.6	Hydrolase activity acting on glycosyl bonds hydrolase activity alpha, alpha-trehalase activity	Cellular hyperosmotic response metabolic process cellular response to DNA damage stimulus trehalose catabolic process trehalose metabolic process	Periplasmic space outer membrane-bounded periplasmic space	Cardona et al., 2010
Protein of sialidase A *Streptococcus pneumoniae*	2vvz (2.50 Å) 113.69 kDa	−357.7	Exo-alpha-sialidase activity	Carbohydrate metabolic process	Membrane	Xu et al., 2008
Protein of vitamin B12 transporter BtuB *Escherichia coli*	2gsk (2.10 Å) 78.07 kDa	−354.4	Vitamin transmembrane transporter activity metal ion binding protein domain specific binding porin activity protein binding	Vitamin transmembrane transport ion transmembrane transport cobalamin transport ion transport	Pore complex intrinsic component of cell outer membrane integral component of membrane membrane cell outer membrane	Shultis et al., 2006
Cellobiohydrolase *Clostridium thermocellum*	1rq5 (2.40 Å) 69.45 kDa	−352.7	Cellulose binding cellulase activity metal ion binding	Enzymes that hydrolyse O- and S-glycosyl compounds polysaccharide catabolic process	Extracellular region	Schubot et al., 2004
Pectate lyase protein *Bacillus subtilis*	2bsp (1.80 Å) 45.56 kDa	−352.1	Pectate lyase activity metal ion binding	Pectin catabolic process	Extracellular region	Pickersgill et al., 1998

*The first column shows the protein name and its source organism (italicized)*.

When considering the biochemical functions of the proteins with the most hydrophobic cavities, we can see that most of them are responsible for selective and non-covalent interaction between identical proteins (identical protein binding), with any proteins or complexes, even containing non-protein molecules (protein binding), with chloride ions (Cl^−^) (chloride ion binding), with any metal ion (metal ion binding), or with anions, charged atoms or groups of atoms with negative net charge (anion binding). The exceptions from this observations are the pre-protein translocase secY subunit from *M. jannaschii* and ammonium transporter from *A. fulgidus* which enable protein transfer across cell membrane (protein transmembrane transporter activity) without specific binding function. Proteins from this group are generally responsible for transport phenomena. For example, the ammonia channel protein from *E. coli* catalyze the transport of single molecular species across the membrane (uniporter activity). Such transport is independent of the movement of any other molecular species. Some proteins enable active transport of a solute across the membrane by a mechanism whereby two or more species are transported together in the opposite directions in a tightly coupled process. Such process does not have to be directly linked to a source of energy other than chemiosmotic energy (antiporter activity). A similar process where molecular species are transported in the same direction (symporter activity) is also enabled by one of the considered proteins—proton glutamate symport protein from *P. horikoshii*. The proteins considered here enable also the cross-membrane transfer of ammonium (ammonium transmembrane transporter activity), glutamate (glutamate: sodium symporter activity), L-aspartate—anion from aspartic acid (L-aspartate transmembrane transporter activity), other amino acids (amino acid: sodium symporter activity), chloride ions (chloride transmembrane transporter activity), and the transmembrane transfer of a chloride ion by a voltage-gated channel (voltage-gated chloride channel activity).

From the biological point of view, the selected proteins with the most hydrophobic cavities are responsible for transport of various structures, such as ions (nitrate, chloride, ammonium, etc.), carbon dioxide, inorganic anions or even amino acids or proteins from, to or between cells across the membrane. Ammonia channels protein is also responsible for processes that form an integrated mechanism by which a cell detects the depletion of primary nitrogen source, usually ammonia, and then activates genes to scavenge the last traces of the primary nitrogen source and to transport and metabolize alternative nitrogen sources. The proteins from this group are embedded within the phospholipid bilayer. In summary, proteins with the most hydrophobic cavities are usually responsible for binding and molecular transport processes.

By inspecting the 10 proteins with the most hydrophilic cavities listed in Table 4, we infer that five of them are membrane components. The iron(III) dicitrate transport protein Feca from *E. coli* and ferripyoverdine receptor from *P. aeruginosa* are signaling proteins. Moreover, from the biological point of view they are responsible for iron ion or siderophore transport. This means that they keep the iron ion homeostasis constant. Similarly, the protein of vitamin B12 transporter BtuB *E. coli* is responsible for the ion and vitamin transmembrane transport. Next, we have also six proteins that are responsible for catalytic processes. Two of them, protein of periplasmic trehalase from *E. coli* and protein of sialidase A from *Streptococcus*, are also membrane proteins. From the biological point of view, they participate in catabolic and metabolic processes. The next two proteins, phenol hydroxylase component from *P. stutzeri* and *O. aries* (sheep) lactoperoxidase are assigned to the extracellular region. They are necessary for oxidation-reduction processes. Moreover, the sheep lactoperoxidase protein plays a role in metal ion binding. The last two of the catalytic proteins, cellobiohydrolase from *C. thermocellum* and pectate lyase protein from *B. subtilis* are responsible for metal ion binding, but their most important functions are the cellulase and pectate lyase activities, thus they are responsible for catabolic processes. The last protein from this group is a protein from adeno-associated virus. This protein is different from the proteins described above, as a component of viral capsid, but it is still related to a membrane-like behavior because it is responsible for permeabilization of host organelle membrane, and then it is involved in the viral entry into host cell.

Our results obtained with a smaller accuracy are comparable to the precise results of cavity volume calculations in case of the PR-10 proteins presented in our previous work (Chwastyk et al., 2016). Our selection of the proteins with cavities, and pockets is different than study (Gao and Skolnick, 2013) of structures deposited in the PDB that is based on protein-ligand binding and structural comparison methods. We provide a new definition of a pocket which is more precise in comparison to just a “ligand binding site” (Gao and Skolnick, 2013). Moreover, we add informations about chemical properties of the pockets considered in that paper.

**Figure 8:**
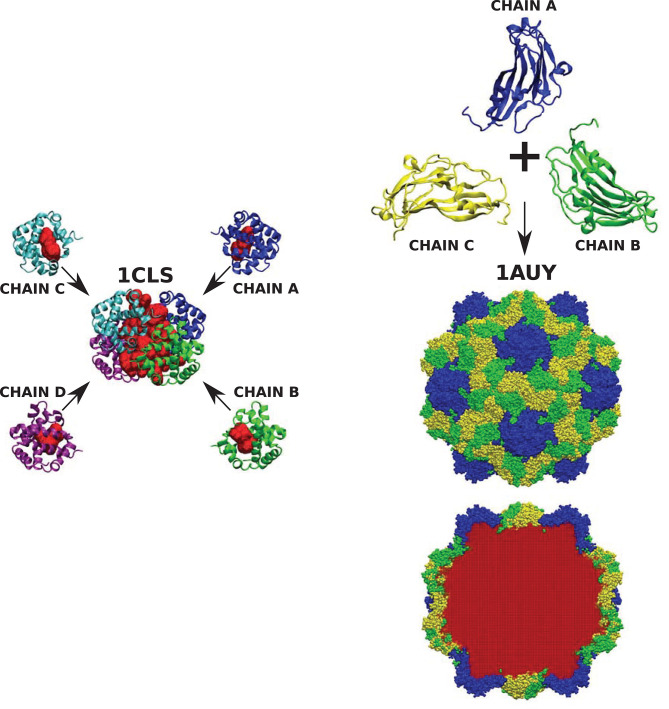
(Left) Cross-linked human hemoglobin (HbA) with each subunit of the tetramer colored differently. Each protein chain contains one cavity (marked red) of size 0.734 ± 0.009, 0.722 ± 0.014, 0.712 ± 0.007, and 0.694 ± 0.012 nm^3^, respectively. The volume of the cavity in the tetrameric hemoglobin structure (marked red) equals 7.634 ± 0.129 nm^3^. (Right) The structure of turnip yellow mosaic virus (TYMV) (in the center) and its component chains (top). None of them protein chains has a cavity, but they create a structure of the virus capsid which encloses a cavity (marked in red at the bottom) of size 6731.10 ± 99.12 nm^3^.

We emphasize that the results have been obtained from the analysis of single chains of various CATH proteins. We should point out that cavities often appear not only within single protein subunits but also within complete quaternary structures. One such example is cross-linked human hemoglobin (HbA) presented in Figure 8. The full quaternary structure with a central cavity measuring 7.634 ± 0.129 nm^3^ is composed of four chains each containing smaller cavities that are an order of magnitude smaller. A similar situation can be observed in more complex structures, like the capsid of the turnip yellow mosaic virus (TYMV) which is formed from of three different protein subunits. None of them contains any cavity. The volume of the cavity within the virus capsid, however, is 6731.10 ± 99.12 nm^3^.

## 5. Conclusions

**Figure 9:**
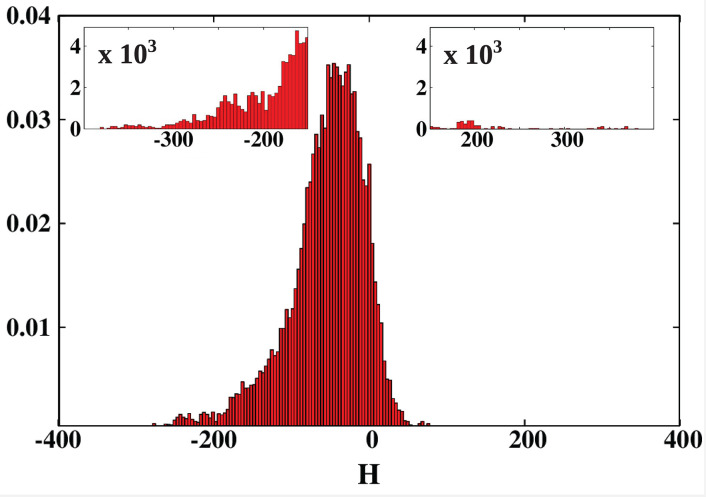
Histogram of the hydropathy index H of all examined structures. The insets zoom on the histogram tails. The most hydrophobic proteins, present in the positive tail, are listed in Table 3 and are marked in green in Figures 2, 5–7. The most hydrophilic proteins (negative tail of the histogram) are listed in Table 4 and are marked in blue in Figures 2, 5–7. The histogram shows that most of the cavities in proteins deposited in the PDB are hydrophilic.

We conducted a survey of 24,280 protein structures from the CATH database. For each of the considered structures we calculated the net hydropathy index. The results are presented as a histogram in Figure 9. The most surprising result is that, unlike in the PR-10 proteins, most of the cavities are hydrophilic. Moreover, the largest cavities are also hydrophilic. On the other hand, the smallest cavities (in small proteins) are highly hydrophobic.

## Data Availability Statement

The raw data supporting the conclusions of this article will be made available by the authors, without undue reservation.

## Author Contributions

MCh, MJ, and MCi designed the research. MCh, EP, and JM performed the research. MCh, EP, JM, MJ, and MCi analyzed the data. MCh and JM created the SPACEBALL website. MCh, EP, MJ, and MCi wrote the paper. MJ and MCi supervised the research. All authors contributed to the article and approved the submitted version.

## Conflict of Interest

The authors declare that the research was conducted in the absence of any commercial or financial relationships that could be construed as a potential conflict of interest.
